# Utilizing Multi-omics analysis to elucidate the role of mitochondrial gene defects in Gastric cancer progression

**DOI:** 10.1371/journal.pone.0325520

**Published:** 2025-06-09

**Authors:** Jie Chu, Hanying Song, Kemin Fu, Wei Xiao, Jiudong Jiang, Qixin Gan, Bo Deng

**Affiliations:** 1 Cancer Center, Ziyang Central Hospital, Ziyang, China; 2 Department of Radiation Oncology, The Sixth Affiliated Hospital, Sun Yat-sen University, Guangzhou, Guangdong, China; 3 Department of Radiology, The First Affiliated Hospital of Hunan College of Traditional Chinese Medicine(Hunan Province Directly Affiliated TCM Hospital), Lusong District, Zhuzhou City, Hunan Province, People's Republic of China; Shanghai University of Traditional Chinese Medicine, CHINA

## Abstract

**Background:**

Gastric cancer is a leading cause of cancer-related mortality worldwide, with poor survival outcomes despite advances in diagnostic and therapeutic methods. Mitochondrial autophagy, or mitophagy, is crucial for maintaining cellular homeostasis and has significant implications in tumor biology. DUSP1, a bispecific phosphatase regulating MAP kinase activity, has been associated with various cancers, but its role in GC remains unclear.

**Materials and methods:**

In order to gain a deeper understanding of gastric cancer cells, this study utilized bulk RNA-seq data from TCGA and GEO, combined with the MSigDB database, to screen for mitophagy-related genes. Univariate Cox regression and LASSO analysis were employed to further identify key mitophagy-related genes. Single-cell RNA sequencing data from the database was analyzed using Seurat software to investigate the mitochondrial autophagy genes in each candidate gastric tissue. To clarify the functional pathways involved, enrichment analysis and differential gene expression analysis were conducted. The characteristics of the immune microenvironment were assessed using the CIBERSORT R package. Additionally, both the ssGSEA algorithm and the CIBERSORT algorithm were utilized to evaluate changes and effects in immunological characteristics during gastric cancer pathogenesis.

**Results:**

We identified eight prognostic genes—STX10, CDC37, VPS35, RCAN1, TRIM25, DUSP1, SEC23A, and GLT8D1—using LASSO-Cox regression analysis. RCAN1 and DUSP1 are strongly positively correlated, while DUSP1 is strongly negatively correlated with TRIM25, and CDC37 is strongly negatively correlated with SEC23A. By incorporating mitochondrial autophagy scores and clinical characteristics, we established a prognostic model that accurately predicts the 3-year survival status of gastric cancer (GC) patients. Additionally, our single-cell analysis identified DUSP1 as a key mitophagy-related gene. Functional studies demonstrated that DUSP1 knockdown significantly inhibits GC cell proliferation and migration.

**Conclusion:**

In this study, we developed a risk score based on eight mitochondrial autophagy-related genes and analyzed their expression across different cell types using single-cell analysis. DUSP1 stood out as a key player in gastric cancer progression, with higher expression in tumor tissues and a significant role in cell proliferation, apoptosis, and drug resistance. Our research also linked this risk score to tumor microenvironment immune cell infiltration and tumor mutational burden, revealing distinct high and low-risk groups in gastric cancer patients. This risk score holds potential for improving patient survival assessment and guiding personalized treatment, including enhancing immunotherapy efficacy.

## Introduction

Gastric cancer is a fatal disease with a low survival rate worldwide. It is reported that there are over one million new cases every year, and gastric cancer is the fifth most common diagnosed malignant tumor globally [1]. Although the past 20 years had been characterized by the expansion of clarifying the molecular mechanism of Gastric Carcinoma(GC) and an advance in diagnostic and therapeutic methods for managing GC patients, the survival outcomes have remained poor [2]. Mitophagy is an extremely important cellular event, which is required for the regulation of cellular environmental homeostasis. As an important regulatory mechanism for cells to remove damaged mitochondria and maintain internal and external balance, mitophagy has significant impacts in the occurrence, progression and treatment of tumours [3]. Dual Specificity Phosphatase 1(DUSP1) is a bispecific phosphatase that regulates Mitogen Activated Protein (MAP) kinase activity. Studies have found that the absence of DUSP1 expression is associated with certain cancers [4]. In several human epithelial tumors, elevated levels of DUSP1 have been reported, including in prostate, colon, and bladder cancer. However, the expression of DUSP1 in tumors progressively decreased with a higher histological grade, indicating that the function and mechanism of DUSP1 in tumors may vary and is complex [5]. In various human cancers, abnormal expression of DUSP1 was observed which was associated with prognosis of tumor patients. Further studies have revealed its role in tumorigenesis and tumor progression [6]. In summary, these studies suggest that abnormal expression of mitochondrial autophagy related genes may lead to GC progression and affect the prognosis of GC patients.

In our current research, we investigated the correlation between mitochondrial autophagy related genes and GC progression. We divided the obtained data into a tumor group and a control group for comparison, and the results showed that there were 22 mitochondrial autophagy related genes with differential expression, and most of the genes were upregulated in the tumor group. In cancer risk assessment, mutation identification is also a crucial step, which may help develop prevention or treatment strategies for GC [7]. Based on somatic mutation data from TCGA-STAD(Stomach adenocarcinoma, STAD) patients, we further investigated the mutation patterns of mitochondrial autophagy related gene features. We found that Missense mutation is widely present in gastric cancer patients. After extracting mutation data of mitochondrial autophagy related genes, it was found that most genes had undergone mutations, indicating that mitochondrial autophagy genes may affect function and mechanism through mutations, thereby affecting the progression of gastric cancer.

To identify mitochondrial autophagy related genes associated with STAD survival outcomes, we screened eight prognostic genes (STX10, CDC37, VPS35, RCAN1, TRIM25, DUSP1, SEC23A, GLT8D1) through LASSO-Cox regression analysis. RCAN1 and DSUP1 are strong positive correlation, while DSUP1 and TRIM25, CDC37 and SEC23A have a strong negative correlation. Subsequently, we analyzed the immune related characteristics of the high and low-risk groups. This analysis indicates that the high and low-risk groups exhibit different types of immune cell infiltration, and patients with high TMB exhibit enhanced response after receiving immune checkpoint blockade therapy, which can lead to long survival and sustained clinical benefits. In addition, we constructed a prognostic model based on mitochondrial autophagy score and clinical pathological characteristics of gastric cancer patients, and evaluated the clinical predictive performance of the model. The results showed that the model can predict gastric cancer survival status, and the model has the best clinical predictive performance for 3 years, and the model has the best clinical predictive performance for 3 years. Therefore, mitochondrial autophagy related genes play a crucial role in the development of GC tumors and may have clinical value as potential biomarkers.

In summary, we have developed and validated mitochondrial autophagy related gene features for prognostic stratification in GC patients. In addition, the constructed prognostic model has clinical practicality and can be used to predict the survival and treatment response of GC patients during treatment. However, it is worth noting that our current research still has certain limitations. In the future, it is necessary to determine the role of mitochondrial autophagy and the key regulatory components of each mitochondrial autophagy pathway in tumor progression, in order to develop precise medical methods targeting specific components or key regulatory factors of mitochondrial autophagy to inhibit the progression of GC.

## Methods

### Data download

The approval was reviewed by the Ethics Committee of Ziyang Central Hospital (No. 269, 2022). All participants provided informed consent. For written consent, participants provided written informed consent. For verbal consent, the consent process was documented by a witness or a research team member, and records were kept. All our data processing began on July 28, 2022. The gastric cancer patient expression profile data, encompassing 407 samples, were retrieved from the official TCGA GDC website (https://portal.gdc.cancer.gov/). This dataset comprises both count and FPKM values. Within this dataset, there are 32 normal samples denoted as 11A, The remaining 375 samples correspond to tumor tissues designated as 01A. Their basic data are in Table. Additionally, clinical data for 470 patients were obtained, which encompassed information such as gender, age, TMN stage, and survival prognosis. Following rigorous screening, a subset of 350 patients were retained for subsequent analysis, ensuring that all pertinent clinical information was included. The somatic mutation data, consisting of 431 records, were obtained and designated as “Masked Somatic Mutation. ” These data were utilized for downstream analysis, specifically employing R’s maftools [8] package to visualize the somatic mutation landscape. This analysis allowed for the calculation of the tumor mutation burden (TMB) for each patient, providing insights into the mutational landscape of the cohort. Furthermore, the microsatellite instability (MSI) data for patients within the TCGA-STAD dataset were acquired from published literature (https://www.cbioportal.org) [9], supplementing the comprehensive dataset and enabling a more holistic exploration of gastric cancer biology.

The gene expression data for GSE844373 [10] and GSE154594 [11] were retrieved from the GEO database, and clinical data specifically for GSE84437 were obtained. All data samples were sourced from Homo sapiens. The GSE15459 chip platform is based on GPL570 [HG-U133_Plus_2] Affymetrix Human Genome U133 Plus 2. 0 Array, while the GSE84437 chip platform is founded on GPL6947 [Illumina HumanHT-12 V3. 0 expression beadchip]. Patients with missing clinical information were systematically excluded from the analysis, resulting in a total of 433 STAD tissue samples from GSE84437 and 200 gastric cancer tissue samples from GSE88770, all of which were meticulously included in the subsequent analysis. To ensure rigorous analysis, each dataset was independently normalized using R’s limma package, ensuring that the gene expression profiles were suitably prepared for downstream investigations. This normalization step is essential for minimizing potential technical biases and ensuring that the datasets can be effectively compared and integrated for comprehensive analyses.

### Acquisition of mitophagy-related gene list and differential analysis

The GeneCards database (http://www.genecards.org/) [12] is a comprehensive resource that consolidates gene-centric information from approximately 150 diverse web sources, encompassing genomic, transcriptional, protein biology, genetic, clinical, and functional data. In our investigation, a search for “mitophagy” within this database yielded a collection of mitophagy-related genes. We proceeded to select genes exhibiting a correlation score surpassing 0. 9 for subsequent analysis. Additionally, we acquired the mitophagy-related gene sets, denoted as “REACTOME_MITOPHAGY,” from Molecular Signatures Database V7. 0 (MSigDB) [13], comprising 29 gene sets. After careful integration and removal of duplicates, a final list of 489 mitophagy-related genes was compiled, with comprehensive details available in Table S1. To further our analysis, we leveraged the grouping information within the dataset and utilized the R package limma to perform differential gene expression analysis. In this analysis, genes with a logFC (log-fold change) greater than 1 and a P-value less than 0. 05 were identified as up-regulated differentially expressed mitophagy-related genes, indicating an increase in expression. Conversely, genes with a logFC less than −1 and a P-value less than 0. 05 were identified as down-regulated differentially expressed mitophagy-related genes, signifying a decrease in expression. This rigorous approach allowed us to pinpoint and categorize genes exhibiting significant expression changes related to mitophagy.

### Univariate cox regression and LASSO analysis

To assess the predictive potential of mitophagy gene expression in gastric cancer, we employed a two-step approach involving univariate Cox regression analysis and Least Absolute Shrinkage and Selection Operator (LASSO) regression analysis using the TCGA-STAD dataset. Firstly, we conducted univariate Cox regression analysis to quantify the association between mitophagy genes and gastric cancer. In this analysis, genes with a statistical significance represented by a P-value less than 0. 05 were retained as candidates that exhibit potential predictive power for gastric cancer. Subsequently, we employed the glmnet package [14] for dimensionality reduction using the LASSO algorithm. LASSO helps identify and select meaningful variables from the candidate genes identified in the previous step. It shrinks the coefficients of less informative genes to zero, effectively reducing the dimensionality of the dataset and retaining only the most relevant genes for building a predictive model. By combining these two analytical approaches, we aimed to construct a robust predictive model for gastric cancer based on mitophagy gene expression, optimizing both feature selection and model performance. This approach is valuable for identifying key genes that may serve as potential biomarkers for gastric cancer prediction. The calculation formula of the risk score is as follows: risk Score = $\begin{array}{c} \sum {\mathrm{\backslash nolimits}}_{i}Coefficient\;\left({gene}_{i}\right)*mRNA\;Expression\;({gene}_{i}) \end{array}$.

### GO/KEGG enrichment analysis of differentially expressed genes in high and low risk groups

Gene Ontology (GO) [15] enrichment analysis is a common method employed for investigating large-scale functional enrichment of genes across various dimensions and levels. Typically, this analysis spans three primary categories: Biological Process (BP), Molecular Function (MF), and Cellular Component (CC) [16]. In addition to GO analysis, the Kyoto Encyclopedia of Genes and Genomes (KEGG) [17] database serves as a valuable resource, containing comprehensive information related to genomes, biological pathways, diseases, and drugs. For the analysis of differentially expressed mitophagy-related genes, we utilized the R package clusterProfiler [18,19]. This package facilitates GO functional annotation, aiding in the identification of significantly enriched biological processes. The results of this enrichment analysis were then effectively visualized using the R package GOplot [20]. To determine statistical significance, a significance threshold for the enrichment analysis was established, with a criterion set at P < 0. 05. This threshold ensures that only enriched biological processes with a statistically significant association with the differentially expressed mitophagy-related genes are considered in the analysis, providing valuable insights into the functional implications of these genes in the context of gastric cancer.

### GSEA enrichment analysis of differentially expressed genes in high and low risk groups

Gene Set Enrichment Analysis (GSEA) is an enrichment method introduced by the Broad Institute, and it offers both the associated analysis software [21,22], and a gene set database called MSigDB. GSEA is primarily employed to determine whether a predefined set of genes exhibits a statistically significant difference in expression between two distinct biological states. It is commonly used to assess alterations in pathway and biological process activity within expression datasets. To explore disparities in biological processes between two sample groups based on gene expression profiles, we leveraged the reference gene sets “c5. go. v7. 4. entrez. gmt” and “c2. cp. kegg. v7. 4. entrez. gmt. ” These gene sets serve as a foundation for enrichment analysis and visualization of the datasets. This analysis was conducted using the GSEA method, which is incorporated into the R package “clusterProfiler. ”In this analysis, statistical significance was determined by setting a threshold of P-value < 0. 05. Results meeting this criterion were considered statistically significant, thereby highlighting the biological processes and pathways that exhibit significant differences between the two groups of samples. This approach enables a comprehensive understanding of the functional distinctions underlying the gene expression profiles in the context of the study.

### GSVA enrichment analysis of differentially expressed genes in high and low risk groups

Gene Set Variation Analysis (GSVA) is a non-parametric and unsupervised analysis technique used to assess gene expression patterns across different samples [23]. It transforms the gene expression matrix into a gene set expression matrix, facilitating the evaluation of gene set enrichment in transcriptomic data. This approach is particularly useful for detecting variations in biological processes among different sample groups. To investigate the variability in biological processes between the two groups of samples, we employed the R package GSVA [23]. We also retrieved the reference gene set “c2. all. v7. 5. 1. symbols. gmt” from the MSigDB database. Using these resources, we calculated the enrichment score for each sample within each pathway in the dataset. To identify pathways that exhibited statistically significant differences, we integrated the R package limma [24]. This allowed us to perform a thorough screening of pathways with notable distinctions between the sample groups. For visualization purposes, the GSVA enrichment results were presented using a heatmap, a graphical representation that provides a visual overview of the pathway enrichment patterns. In this analysis, statistical significance was determined using a threshold of P-value < 0. 05, ensuring that only pathways with substantial differences in enrichment were considered statistically significant. This comprehensive approach aids in uncovering and understanding the underlying variations in biological processes across the sample groups.

### CIBERSORT

CIBERSORT (https://cibersort.stanford.edu/) is a valuable R/web tool designed for deconvoluting the expression matrix of human immune cell subtypes [25,26]. It operates on the principles of linear support vector regression and can assess the infiltration status of immune cells within sequencing samples, leveraging a gene expression signature set encompassing 22 known immune cell subtypes. In our study, the CIBERSORT algorithm played a pivotal role in evaluating the infiltration status of immune cells within the gastric cancer dataset. Subsequently, we utilized the Wilcoxon test to examine the differences in immune cell infiltration between gastric cancer samples and normal samples. A significance threshold of P < 0. 05 was established, indicating that statistical significance was attributed to differences in immune cell infiltration. This analysis provided insights into the immune microenvironment of gastric cancer, highlighting potential differences in immune cell composition between tumor and normal tissues.

### Prognostic correlation analysis of different risk factors in gastric cancer

We select clinical indicators (age, gender, risk score) commonly used in the prognosis of gastric cancer. Among these possible prognostic factors, risk score and age are used as continuous variables, and gender is converted into a categorical variable. The results are drawn into a forest plot with the forestplot package, showing Odds ratios and P-values for each factor. We then use the nomogram package [27] to express the relationship between variables in the prediction model, and use the DCA (Decision Curve Analysis) curve to predict the model evaluation effectiveness of 1 year, 3 years, and 5 years.

### Singer cell analysis

Single-cell data of gastric cancer were obtained from the publicly available dataset GEO (GSE167297). The 10 × Genomic platform sequenced the dataset. For analysis using the R package Seurat, we filtered cells with Unique Molecular Identifier (UMI) counts less than 200, while mitochondrial genes > 20% also be filtered [28]. The integrated data were screened for high variant genes, while the high variant genes were regioncentric using the ScaleData function. The data are then subjected to PCA analysis and clustering analysis using the FindNeighbors process.

### Immunohistochemistry

Paraffin-embedded tumor tissue was cut into 4 μm sections, dewaxed, and rehydrated. Immunohistochemical staining was performed according to standard protocols. Immunohistochemical staining: After slicing, dewaxing and antigen repair were performed, and the normal sheep serum working solution was sealed. Each tissue was dripped with DUSP1 Polyclonal Antibody (protentech, 16112–1-AP) and incubated overnight at 4 °C in a refrigerator. Incubate the secondary antibody for 30 min after cleaning. Then, perform color rendering, re-staining, dehydration, and sealing. Finally, take photos using a microscope (Nikon, ECLIPSE Ts2-FL).

### Cell culture, shRNA transfaction and RTqPCR

The human cell line HGC27 and AGS were purchased at Cellcook (https://www.cellcook.com/) and provided for genotyping identification. The HGC27 and AGS were individually cultured in DMEM and 1640(Gibco, C11995500BT) containing 20% (v/v) fetal bovine serum (FBS, ExCell Bio, FSP500) and 1% penicillin–streptomycin (penicillin 100 U/ml and streptomycin 0. 1 mg/ml, Beyotime). And the cells were incubated at 37 °C in a 5% CO2 incubator. The shRNA against DUSP1 and negative control (NC) were ordered in Beijing Tsingke Biotech (https://tsingke.com.cn/). The sequence of the shRNA is as follows: shDUSP1(sense 5′–3′): Forward Primer: TGTTGTTGGATTGTCGCTCCT, Reverse Primer: TTGGGCACGATATGCTCCAG; GAPDH(sense 5′–3′): Forward Primer: GCAGGGGGGAGCCAAAAGGGT, Reverse Primer: TGGGTGGCAGTGATGGCAT. HGC27 and AGS were first spread into 6-well plates, and after growing to 70–80%,the cells were transfected with plasmids by polyethylenimine (PEI, Polysciences, 23966–1). RTqPCR detected the knockdown efficiency of DUSP1. Total RNA was extracted with TRIzol reagent (Takara) and then reversed and transcribed into cDNA using the PrimeScript RT Master Mix kit (Takara, RR036A). RT-PCR was performed using TB Green® Premix Ex Taq™ II(Takara,RR820A). GAPDH was used as a reference for endogenous normalization. (Reverse) GAGCACAGGGTACTTTATTG.

### Cell Counting Kit-8 (CCK-8) assay, plate cloning

The GC cells and the shRNA transfaction cells were plated into clear bottom 96-well plates, 3000 cells per well for 24h, 48h and 72h. After, the medium was replaced with the 100ul culture medium containing 10ul CCK-8 reagent (AR1160, Boster, China) and the cells were incubated for 4h. The cells’ activity was detected by a microplate reader of 450 nm absorbance.

GC cells were placed into six-well plates, about 3,000 cells per well and cultured with a complete medium for 2 weeks. The medium was replaced every 3 days. Cell colonies were fixed with paraformaldehyde and stained with 0. 1% crystal violet for 20 min. Visible colonies were counted for quantification of results.

### Wound healing assay and invasion experiment

For scratch wound assay, the 6-well plate’ back were used marker pen to draw two horizontal lines, GC cells were seeded at a density of 2,000 cells/well in the plate. When the cells reached about 95% confluence, using a 200ul pipette tip to create three scratch for per well. And then the PBS was used to wash the cells for 3 times. Cells were maintained in serum free DMEM medium. Each scratch was photographed under the microscope at 0h, 24h,48h and 72h. The rate of wound-healing was calculated as the following formula: ((original scratch width- scratch width after healing)/ (original scratch width) −1) × 100%.

The migration experiment was performed with Boyden chamber assay (8-μm pore). 20,000 GC cells’ resuspension with free- FBS medium were added to the top chamber and 0. 6 ml supplemented medium was seeded to the lower chamber. After 24h, the chambers were washed with the PBS for three times, using a cotton swab removed the non- migration cells. Cells were fixed with 4% formaldehyde at room temperature for 15 min and then stained with 0. 1% crystal violet for 20 min. Then cells were counted at 100 × magnification under a microscope, which were selected 5 randomly fields. The migration index (%) was calculated using the following formula: Migration index (%) = (average transmembrane number of treatment group/average transmembrane number of control group) ×100.

### Statistical analysis

**Fig 1:**
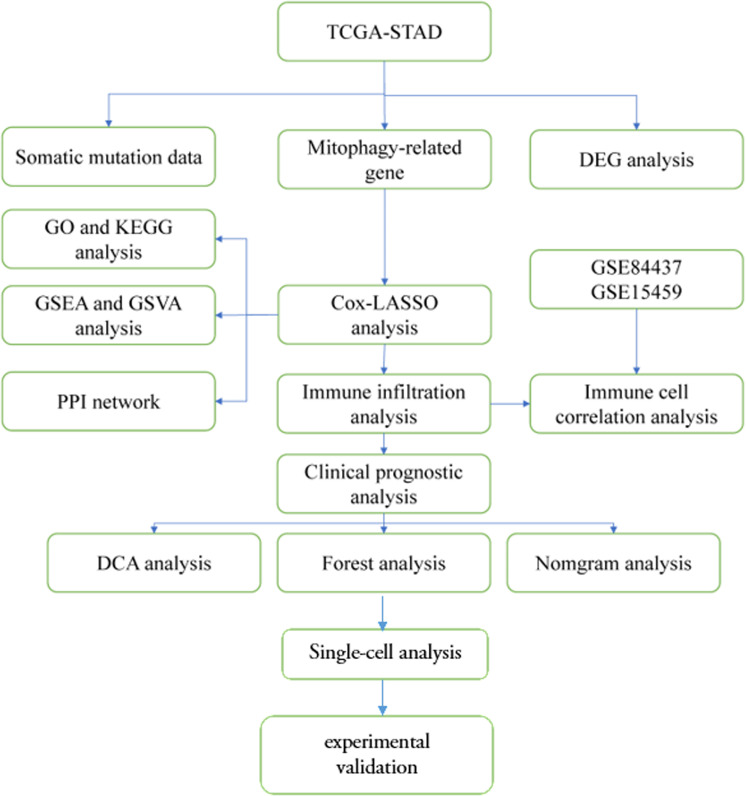
Analysis process.

All data processing and analyses were conducted using R software (version 4. 2. 0). To compare two groups of continuous variables, we employed statistical tests based on the distribution of the variables. Specifically, normally distributed variables were assessed using the independent Student t-test, while non-normally distributed variables were analyzed using the Mann-Whitney U test (Wilcoxon rank sum test). For categorical variables, the statistical significance between two groups was evaluated using either the Chi-square test or Fisher’s exact test, depending on the specific circumstances. Survival analysis was conducted using the “survival” package in R. Kaplan-Meier survival curves were generated to illustrate differences in survival, while the log-rank test was employed to assess the statistical significance of differences in survival time between two groups of patients. All statistical tests were two-sided, and a significance level of P < 0. 05 was deemed statistically significant Fig 1.

## Results

### Analysis process

#### Analysis results of mitophagy genes based on multi-omics.

**Fig 2:**
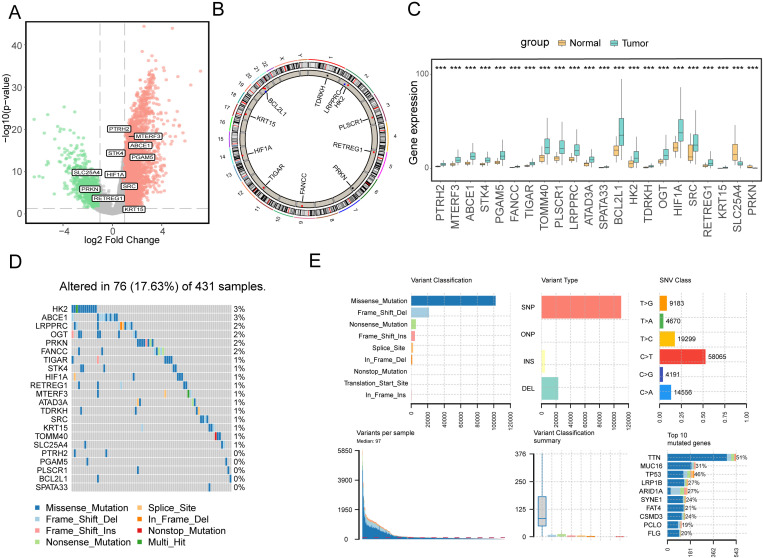
Analysis Results of Mitophagy Genes Based on Multi-Omics. A: The x-axis represents log2FoldChange, and the y-axis represents -log10 (P-value). Red nodes represent up-regulated differentially expressed genes, green nodes represent down-regulated differentially expressed genes, and gray nodes represent genes that are not significantly differentially expressed. B: Chromosomal Location of Mitophagy-Related Genes. C: Comparison of Differential Expression of Mitophagy-Related Genes between the Tumor Group and the Control Group. D: Waterfall Plot Illustrating Mutation Frequency of Mitophagy-Related Genes. E: Comprehensive Overview of Mutations Observed in TCGA-STAD Patients.

In our initial analysis, we conducted a comparative assessment of gene expression using RNA-seq data from TCGA-STAD between the tumor group and the control group. Our investigation revealed significant differences in the expression of 22 mitophagy-related genes, which are listed as follows: PTRH2, MTERF3, ABCE1, STK4, PGAM5, FANCC, TIGAR, TOMM40, PLSCR1, LRPPRC, ATAD3A, SPATA33, BCL2L1, HK2, TDRKH, OGT, HIF1A, SRC, RETREG1, KRT15, SLC25A4, and PRKN. Notably, most of these genes exhibited elevated expression levels in the tumor group, as depicted in Fig 2A and 2C. Furthermore, we conducted a chromosomal location analysis of these 22 mitophagy-related genes using the RCircos package, illustrating their positions on human chromosomes (Fig 2B). Additionally, our analysis of somatic mutation data from TCGA-STAD patients unveiled extensive missense mutations in individuals with gastric cancer. The primary mutation type identified was Single Nucleotide Polymorphism (SNP), with the most common base mutation being C > T. The top 10 mutated genes in this context were TTN, MUC16, TP53, LRP1B, ARID1A, SYNE1, FAT4, CSMD3, PCLO, and FLG (Fig 2C). Furthermore, when we specifically examined the mutation data of mitophagy-related genes, we observed mutations in all genes except PTRH2, PGAM5, PLSCR1, BCL2L1, and SPARA33. Notably, the mutation frequency of HK2 and ABCE1 was 3%, suggesting that mutations may potentially impact the function and mechanisms of mitophagy-related genes (Fig 2D).

#### Protein interaction and regulatory network analysis.

**Fig 3:**
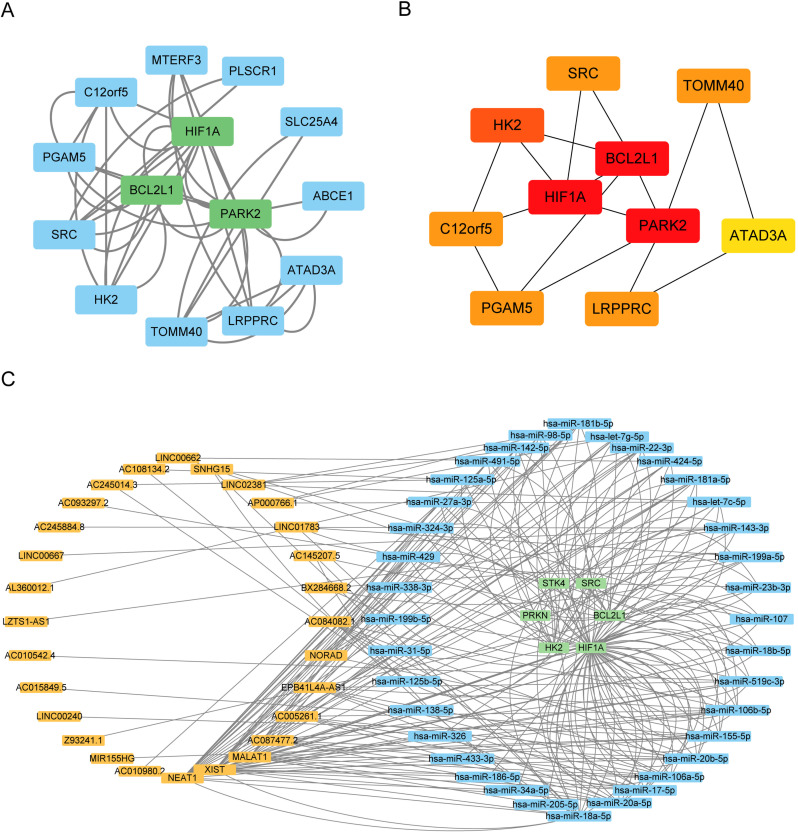
Protein-Protein Interaction (PPI) and ceRNA Regulatory Network Analysis. A. PPI Regulatory Network: This network provides detailed information about the nodes (proteins) within the network. The core gene set identified by MCODE is highlighted with a green box. B. Hub Gene Regulatory Network: This network was generated based on CytoHubba analysis. The intensity of red coloration reflects the CytoHubba score, with darker red nodes indicating higher hub roles within the network. C. ceRNA Regulatory Network: This network represents the competitive endogenous RNA (ceRNA) regulatory relationships. In this network, yellow nodes represent long non-coding RNAs (lncRNAs), blue nodes represent microRNAs (miRNAs), and green nodes represent messenger RNAs (mRNAs). Each color corresponds to a different type of RNA molecule. These networks collectively depict the protein interactions, hub gene roles, and ceRNA regulatory interactions, providing a comprehensive view of the molecular mechanisms underlying mitophagy-related gene regulation.

To identify key hub genes that play pivotal roles in mitophagy, along with their potential molecular interaction mechanisms, we initiated an analysis using the STRING database to investigate protein-protein interaction (PPI) networks. Following a confidence level cutoff of 0. 400, this analysis yielded 14 PPI nodes (proteins) and 84 connecting edges (Table S2). Subsequently, we employed the CytoHubba and MCODE plugins within Cytoscape to further eluc idate interacting proteins that serve as hub genes. Our calculations revealed that HIF1A, BCL2L1, and PARK2 exhibited the highest interaction scores according to both algorithms (Fig 3A and B). Moving forward, we extended our analysis to explore the microRNA (miRNA) molecules and long non-coding RNAs (lncRNAs) that potentially regulate these hub genes. We utilized the mirTarbase database and the ENCORI database for this purpose. Using Cytoscape, we constructed a ceRNA (competitive endogenous RNA) regulatory network to visualize the intricate regulatory relationships among these molecules (Fig 3C). This comprehensive analysis provides insights into key hub genes in mitophagy regulation and their potential regulatory networks involving miRNAs and lncRNAs.

#### Lasso-cox regression analysis to screen prognosis-related genes.

**Fig 4:**
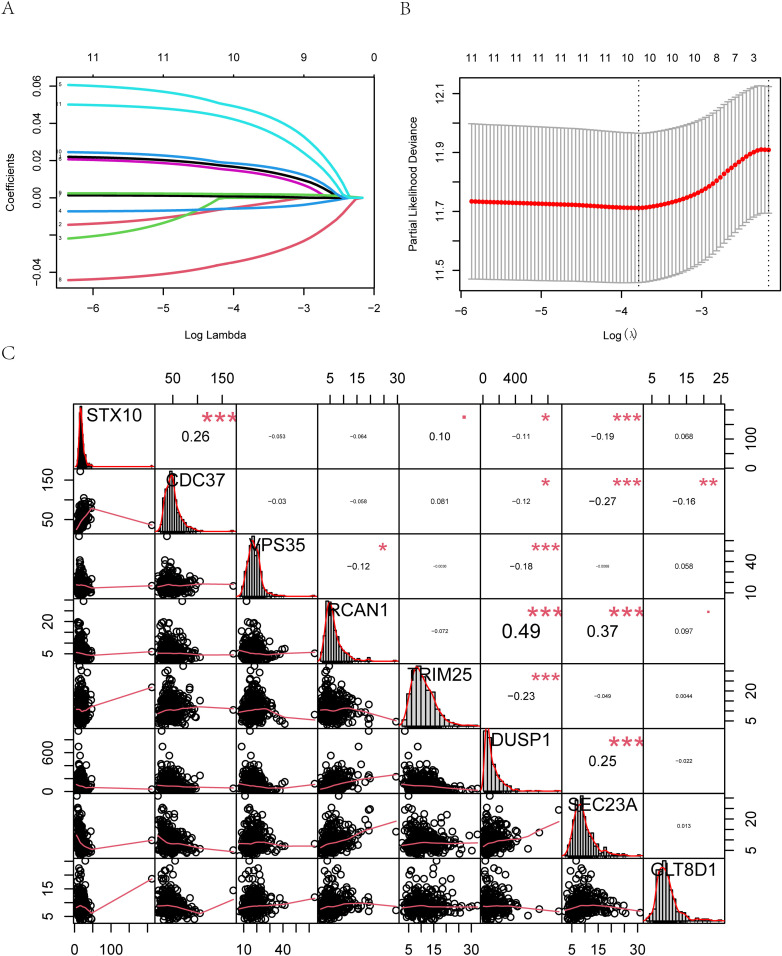
LASSO-Cox Regression Analysis to Screen Prognosis-Related Genes. A. In LASSO-Cox regression analysis, the chart illustrates the correlation (R) for each gene’s contribution to the regression synergy effect. Positive correlations are indicated by R > 0, while negative correlations are represented by R < 0. B. The y-axis shows the evaluation index corresponding to each λ value, with the optimal λ value highlighted. C. Correlation Analysis: Pearson correlation analysis was conducted among the expression values of the 8 mitophagy-related prognostic genes. This analysis reveals the degree of correlation between these genes. Notably, strong positive correlations are observed between RCAN1 and DUSP1 (R = 0. 49), while DUSP1 exhibits a negative correlation with TRIM25 (R = −0. 23), and CDC37 demonstrates a negative correlation with SEC23A (R = −0. 27). (p < 0. 05). These results provide valuable insights into the relationship between the selected genes and their potential influence on prognosis in the context of stomach adenocarcinoma.

In our analysis of transcriptome data from tumor samples in TCGA-STAD, our goal was to identify genes associated with prognostic outcomes in stomach adenocarcinoma (STAD). Specifically, we focused on mitophagy-related genes by compiling a list of 489 candidate genes sourced from both the GeneCards database and the GSEA database (as detailed in Table S1). To pinpoint mitophagy-related genes with prognostic relevance, we employed a series of analytical steps. First, we conducted univariate Cox regression analysis to assess the relationship between survival outcomes and the 489 candidate genes. Subsequently, we applied LASSO-Cox regression analysis to further refine our selection, ultimately identifying 8 prognostic genes with the most optimal parameters. These genes are STX10, CDC37, VPS35, RCAN1, TRIM25, DUSP1, SEC23A, and GLT8D1. Our analysis of the LASSO-Cox regression process is depicted in Fig 4A, which illustrates the Lambda selection, and Fig 4B, which displays the coefficients of the selected genes. Additionally, Fig 4C provides insight into the correlation analysis results of these nine prognostic genes. Notably, RCAN1 and DUSP1 exhibit a strong positive correlation (R = 0. 49), while DUSP1 and TRIM25 (R = −0. 23) as well as CDC37 and SEC23A (R = −0. 27) demonstrate strong negative correlations (p < 0. 05). These findings shed light on the prognostic significance of these mitophagy-related genes and their potential interactions within the context of STAD.

#### Prognostic analysis of the screened mitophagy-related genes.

**Fig 5:**
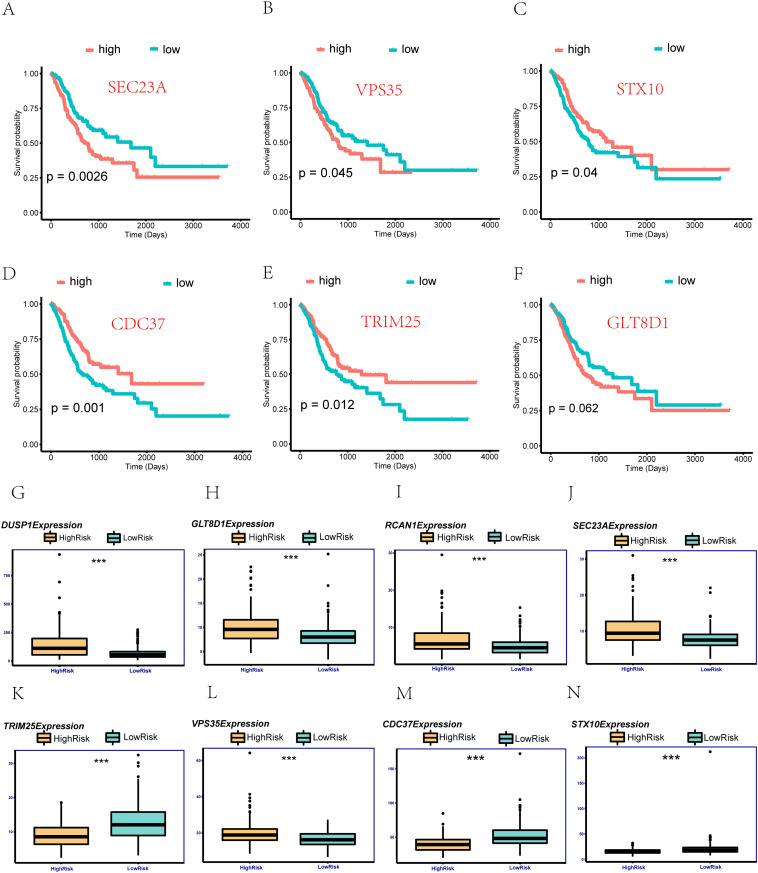
Prognostic Analysis of the Screened Mitophagy-Related Genes. A: Based on the median expression of SEC23A, patients were divided into high and low expression groups, and survival curve analysis was performed. B: Similarly, based on the median expression of VPS35, survival curve analysis was conducted. C: For STX10, patients were categorized into high and low expression groups, followed by survival curve analysis. D: CDC37 expression levels were used to segregate patients into high and low expression groups for survival analysis. E: TRIM25’s median expression guided the classification of patients into high and low expression groups, followed by survival curve analysis. F: Similarly, for GLT8D1, patients were divided into high and low expression groups, and survival curve analysis was performed. G: Boxplot comparing the expression of DUSP1 between two risk groups in TCGA-STAD. H: Expression comparison of GLT8D1 between two risk groups in TCGA-STAD. I: Expression levels of RCAN1 in two risk groups of TCGA-STAD are depicted. J: Boxplot illustrating the expression of SEC23A in two risk groups of TCGA-STAD. K: Expression comparison of TRIM25 between two risk groups in TCGA-STAD. L: Expression levels of VPS35 in two risk groups of TCGA-STAD are shown. M: Boxplot depicting the expression of CDC37 in two risk groups of TCGA-STAD. N: Expression comparison of STX10 between two risk groups in TCGA-STAD. Note: In the Figs, “ns” indicates no significant difference (P > 0. 05), while asterisks represent statistical significance (*P < 0. 05, **P < 0. 01, ***P < 0. 001, ****P < 0. 0001). These analyses provide insights into the prognostic significance and expression patterns of the screened mitophagy-related genes in stomach adenocarcinoma (TCGA-STAD).

STX10, CDC37, VPS35, RCAN1, TRIM25, DUSP1, SEC23A, and GLT8D1 were divided into high and low expression groups based on the median expression level, and survival analysis was performed (Fig 5A-5F), except for DUSP1, RCAN1 and GLT8D1 (the P-value of DUSP1 and RCAN1 are greater than 0. 1, not shown here), the survival outcomes of the two groups were significantly different in each gene (P < 0. 05) (Fig 5A-5F). Among them, the significant difference P-value between SEC23A and CDC37 is less than 0. 01. Among them, low expression of SEC23A and VPS35 genes showed better survival conditions, while high expression of STX10, CDC37 and TRIM25 genes showed better survival conditions. The risk score of each patient is equal to the sum of the gene expression value multiplied by the multivariate Cox regression coefficient, with the median as the cutoff value, all patients are divided into high risk group and low risk group (High risk and Low risk), and the two groups are compared survival outcomes and the expression levels of 8 genes were used to determine the differences in the expression levels of 8 risk assessment factors between the two groups using the Wilcoxon test, and it was found that the 8 risk assessment factors were significantly different between the high and low risk groups (p < 0. 01) (Fig 5G-5N).

#### Identification of the key mitophagy-related genes dusp1 in single-cell analysis. .

**Fig 6:**
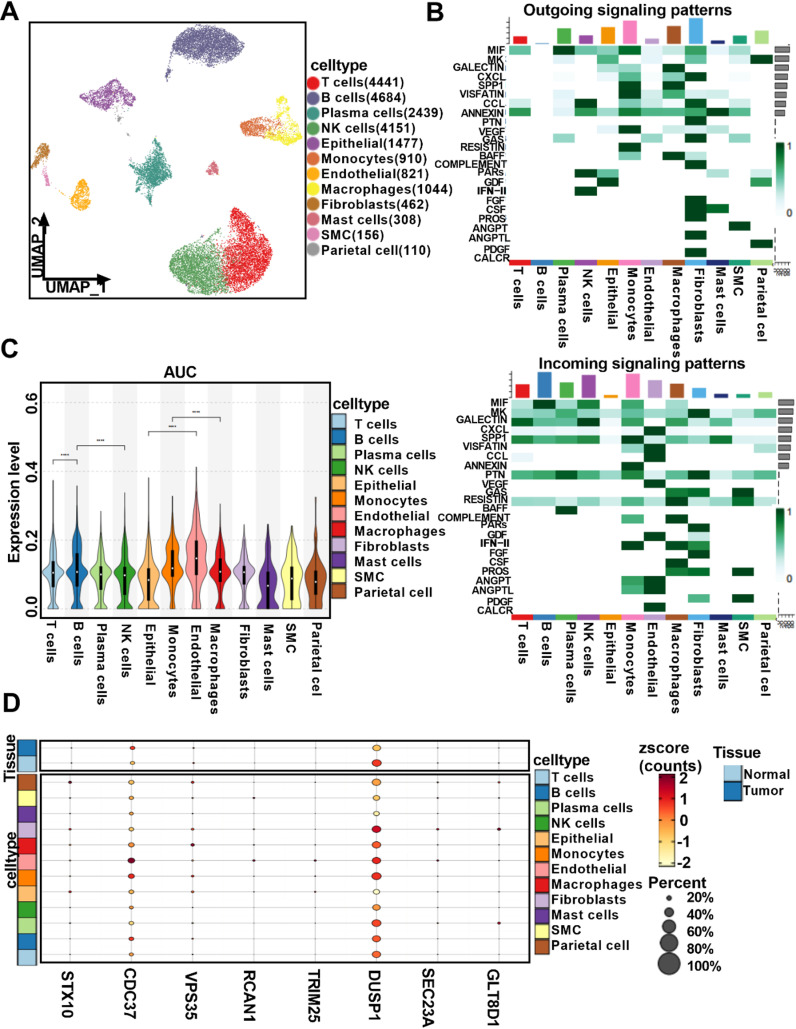
Identification of the role of dusp1 in gastric cancer. (A,C) The expression and distribution of major cell types;(B) cell-cell communication analysis; (D) The distribution of key mitophagy-related genes in many cell types.

To future identification of mitophagy-related genes, our research found that Many kinds of cells types composition was determined, including, T cells, B cells, Plasma cells, NK cells, Epithelial, Monocytes, Endothelial, Macrophages, Fibroblasts, Mast cells, SMC and Parietal cell, which it was strongly correlation with cell-cell communication (Fig 6A, 6B). Subsequently, we also found that mitophagy-related genes were accuracy in T cells, B cells NK cells Epithelial cells, Endothelial cells and Macrophages cell types (Fig 6C). In addition, we also observer that DUSP1 proteins was highly enrichment in many mitophagy-related genes (Fig 6D). Together, among the many key mitophagy-related genes, the DUSP1 was as a key protein leading to progression of gastric cancer.

#### GO/KEGG enrichment analysis of differentially expressed genes in high and low risk groups.

**Fig 7:**
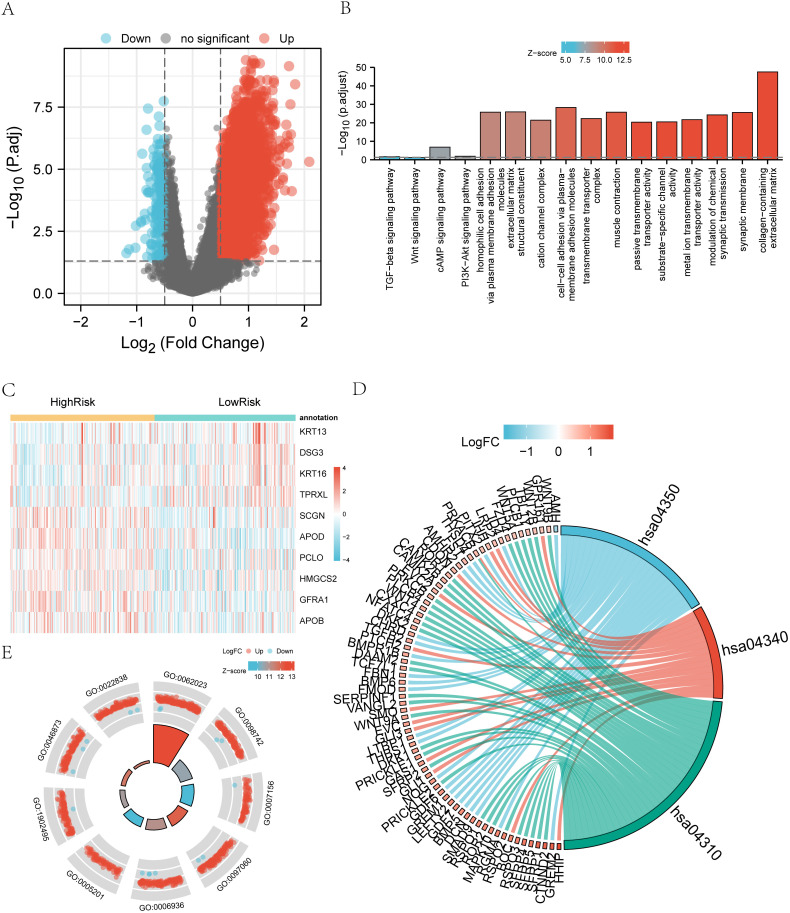
GO/KEGG Enrichment Analysis of Differentially Expressed Genes in High and Low-Risk Groups. A. The Volcano Plot of Differentially Expressed Genes: The x-axis represents log2 fold change, while the y-axis represents -log10 (adjusted P-value). Up-regulated genes are depicted in red, and down-regulated genes are shown in blue. B. Bar Graph of GO and KEGG Enrichment Analysis: The y-axis represents -log10 (P-value), and the x-axis displays enriched GO terms and pathways. C. Heatmap of Differential Gene Expression: The x-axis lists gene names, while the y-axis represents sample groupings. Coloration indicates gene expression levels, with darker red indicating higher expression in the high-risk group and darker blue indicating lower expression. D. Chord Diagram for KEGG Enrichment Analysis: The left portion displays gene color blocks, with different colors representing corresponding logFC values. The right half showcases entry color blocks, with the size of each block indicating the corresponding counts (i. e. , the number of molecules included in this entry in the enrichment analysis). The connecting strings represent molecules contained within the entry. E. Circular Diagram for GO Enrichment Pathways: This diagram is divided into two parts, the inner circle, and the outer circle. Each column in the inner circle corresponds to an entry, with the column height indicating the relative size of p. adj. Higher values represent smaller p. adjust values for the ID. The filled color of each column represents the z-score value associated with the entry. These visualizations provide a comprehensive overview of the GO and KEGG enrichment analysis results, highlighting the significant pathways and gene expression patterns associated with high and low-risk groups in the context of gastric cancer. Additional information on GO enrichment analysis and KEGG enrichment analysis is provided in Table 2 and Table 3.

In our analysis using the limma package, we compared high and low-risk groups within the TARGET dataset. This comparison identified a total of 3,555 differentially expressed genes, with 3,350 genes up-regulated and 205 genes down-regulated (as depicted in Fig 7A). In Fig 7C, the heatmap displays the top 10 genes with the most significant differences, sorted according to their log-fold change (logFC). Among these, 6 genes are up-regulated, while 4 genes are down-regulated. Fig 7B provides an overview of the Gene Ontology (GO) and KEGG enrichment pathways associated with the high and low-risk groups. GO enrichment pathways include Biological Processes (BP) related to gastric cancer pathways such as cell-cell adhesion via plasma-membrane adhesion molecules and homophilic cell adhesion via plasma membrane adhesion molecules. Cellular Component (CC) pathways mainly encompass collagen-containing extracellular matrix and synaptic membrane, while Molecular Function (MF) pathways include extracellular matrix structural constituent and metal ion transmembrane transporter activity. KEGG enrichment pathways predominantly include the cAMP signaling pathway, PI3K-AKT signaling pathway, TGF-beta signaling pathway, and Wnt signaling pathway in relation to gastric cancer. Fig 7E showcases the gene expression of related genes within GO-enriched pathways or biological functions, while Fig 7D illustrates the related genes within KEGG-enriched pathways. These analyses provide insights into the molecular pathways and gene expression changes associated with high and low-risk groups in the context of gastric cancer Table 1.

**Table 1:** Baseline data table.

Characteristic	levels	Overall
n		375
T stage, n (%)	T1	19 (5.2%)
	T2	80 (21.8%)
	T3	168 (45.8%)
	T4	100 (27.2%)
N stage, n (%)	N0	111 (31.1%)
	N1	97 (27.2%)
	N2	75 (21%)
	N3	74 (20.7%)
M stage, n (%)	M0	330 (93%)
	M1	25 (7%)
Age, median (IQR)		67 (58, 73)

**Table 2:** GO analysis.

Description	pvalue	p.adjust
cell-cell adhesion via plasma-membrane adhesion molecules	9.9188E-32	5.9265E-28
homophilic cell adhesion via plasma membrane adhesion molecules	4.2382E-31	1.2662E-27
muscle contraction	1.4782E-29	2.944E-26
modulation of chemical synaptic transmission	2.8381E-28	4.2395E-25
regulation of trans-synaptic signaling	3.7765E-28	4.5129E-25
muscle system process	7.2485E-27	7.2183E-24
synapse organization	1.8399E-26	1.5705E-23
regulation of membrane potential	2.0865E-24	1.5583E-21
synapse assembly	1.1087E-23	7.3608E-21
regulation of blood circulation	6.0353E-23	3.6061E-20
multicellular organismal signaling	9.7198E-23	5.2796E-20
extracellular structure organization	2.3274E-21	1.1588E-18
heart contraction	8.7309E-21	4.0129E-18
extracellular matrix organization	1.7865E-20	7.6245E-18
axonogenesis	2.1483E-20	8.5574E-18
heart process	5.0291E-20	1.8781E-17
regulation of ion transmembrane transport	7.4325E-20	2.6123E-17
regulation of heart contraction	1.0641E-19	3.5323E-17
neuron projection guidance	1.8923E-15	5.9508E-13

**Table 3:** KEGG analysis.

Description	pvalue	p.adjust
Neuroactive ligand-receptor interaction	2.8343E-22	8.8713E-20
Calcium signaling pathway	2.0584E-16	3.2214E-14
Complement and coagulation cascades	1.5216E-13	1.5876E-11
ECM-receptor interaction	1.6799E-11	1.0955E-09
Protein digestion and absorption	1.7499E-11	1.0955E-09
cAMP signaling pathway	3.1985E-09	1.6685E-07
cGMP-PKG signaling pathway	1.258E-08	5.625E-07
Dilated cardiomyopathy	2.4153E-08	9.4497E-07
Circadian entrainment	1.1627E-07	3.7532E-06
Adrenergic signaling in cardiomyocytes	1.3016E-07	3.7532E-06
Arrhythmogenic right ventricular cardiomyopathy	1.319E-07	3.7532E-06
Pancreatic secretion	1.4636E-07	3.8175E-06
Insulin secretion	1.7851E-07	4.298E-06
Axon guidance	2.4746E-07	5.5326E-06
Vascular smooth muscle contraction	2.6693E-07	5.57E-06
Hypertrophic cardiomyopathy	5.9314E-07	1.1603E-05
Salivary secretion	4.2812E-06	7.8824E-05
Gastric acid secretion	4.5492E-06	7.9106E-05
Aldosterone synthesis and secretion	4.9526E-06	8.1587E-05

#### GSEA analysis of differentially expressed genes in high and low risk groups.

**Fig 8:**
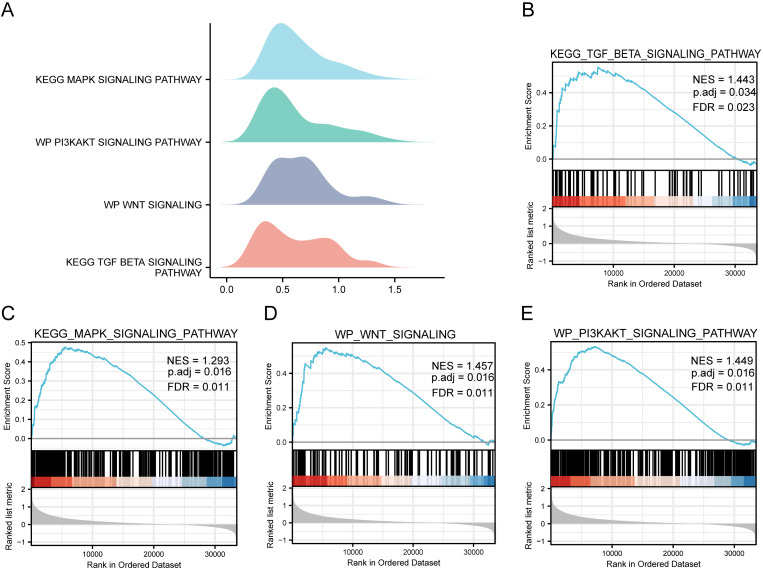
GSEA Analysis of Differentially Expressed Genes in High and Low-Risk Groups. A–E. Hill Plots of GSEA Analysis Results for TCGA-STAD High and Low-Risk Groups. In each plot, the x-axis represents the rank of the gene within the list of differentially expressed genes, with values greater than zero indicating up-regulation and values less than zero indicating down-regulation. The upper y-axis represents the enrichment score, while the lower y-axis shows the log-fold change (logFC) value. Each color corresponds to a specific pathway. Highlighted Pathways: C. MAPK Signaling Pathway (Fig 8C) E. PI3K- AKT Signaling Pathway (Fig 8E).

To investigate the impact of differentially expressed mitophagy-related genes (DEGs) on the development of stomach adenocarcinoma (STAD) between high and low-risk groups in gastric cancer, we conducted Gene Set Enrichment Analysis (GSEA) on the TCGA-STAD dataset. This analysis aimed to reveal the associations between DEGs and biological processes, affected cellular components, and molecular functions. We applied a significance threshold of P < 0. 05 and a false discovery rate (FDR) value (q-value) < 0. 05 for significant enrichment screening. The results of this analysis demonstrated that DEGs between high and low-risk groups in the TCGA-STAD dataset were notably enriched in several signaling pathways, including:A. MAPK Signaling Pathway (Fig 8C) B. PI3K- AKT Signaling Pathway (Fig 8E)

C. TGF-beta Signaling Pathway (Fig 8B) D. Wnt Signaling Pathway (Fig 8D)These findings, illustrated in Figs 8A-8E, underscore the potential involvement of these pathways in the differential expression of mitophagy-related genes and their role in the development of gastric cancer in distinct risk groups.

B. TGF-beta Signaling Pathway (Fig 8B) D. Wnt Signaling Pathway (Fig 8D)These hill plots provide a visual representation of the GSEA analysis results, emphasizing the enrichment of specific pathways in the high and low-risk groups within the TCGA-STAD dataset. Additional information on GSEA enrichment analysis is provided in Table 4.

**Table 4:** GSEA analysis.

Description	pvalue	p.adjust
KEGG_AXON_GUIDANCE	0.000999	0.01580175
KEGG_CALCIUM_SIGNALING_PATHWAY	0.000999	0.01580175
KEGG_CELL_ADHESION_MOLECULES_CAMS	0.000999	0.01580175
KEGG_FOCAL_ADHESION	0.000999	0.01580175
KEGG_MAPK_SIGNALING_PATHWAY	0.000999	0.01580175
KEGG_MELANOGENESIS	0.000999	0.01580175
KEGG_NEUROACTIVE_LIGAND_RECEPTOR_INTERACTION	0.000999	0.01580175
KEGG_REGULATION_OF_ACTIN_CYTOSKELETON	0.000999	0.01580175
KEGG_VASCULAR_SMOOTH_MUSCLE_CONTRACTION	0.000999	0.01580175
NABA_CORE_MATRISOME	0.000999	0.01580175
NABA_ECM_AFFILIATED	0.000999	0.01580175
NABA_ECM_GLYCOPROTEINS	0.000999	0.01580175
NABA_ECM_REGULATORS	0.000999	0.01580175
NABA_SECRETED_FACTORS	0.000999	0.01580175
REACTOME_ADORA2B_MEDIATED_ANTI_INFLAMMATORY_CYTOKINES_PRODUCTION	0.000999	0.01580175
REACTOME_ANTI_INFLAMMATORY_RESPONSE_FAVOURING_LEISHMANIA_PARASITE_INFECTION	0.000999	0.01580175
REACTOME_BIOLOGICAL_OXIDATIONS	0.000999	0.01580175
REACTOME_CARDIAC_CONDUCTION	0.000999	0.01580175
REACTOME_CELL_SURFACE_INTERACTIONS_AT_THE_VASCULAR_WALL	0.000999	0.01580175

#### GSVA analysis of differentially expressed genes in high and low risk groups.

**Fig 9:**
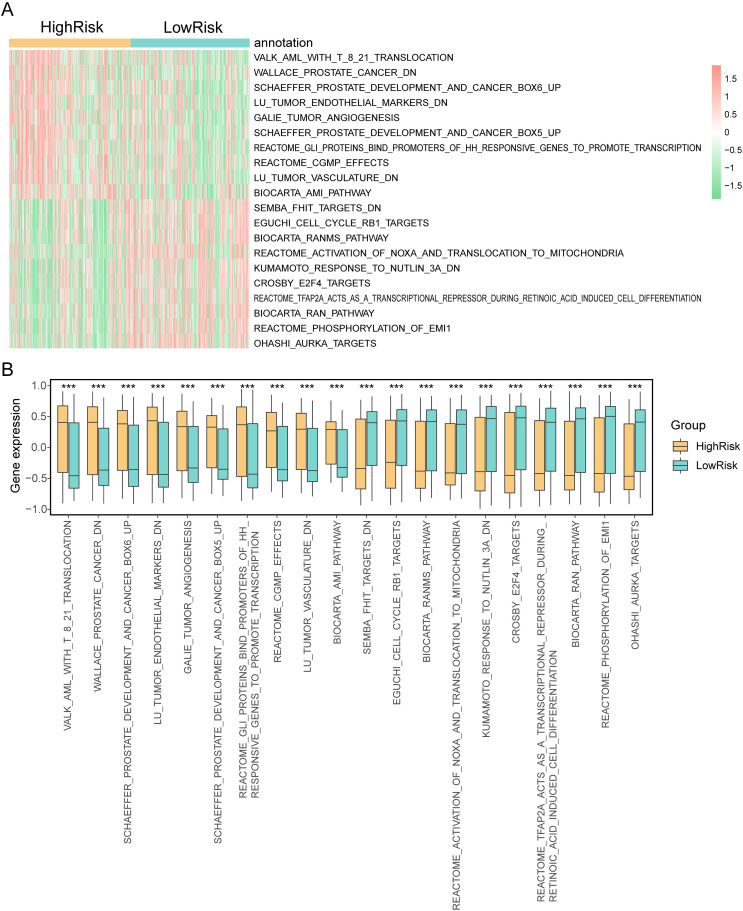
GSVA analysis of differentially expressed genes in high and low risk groups. A-B. The complex numerical heat map (A) of the GSVA enrichment analysis results of DEGs between high and low risk groups in the TCGA-STAD dataset, and the group comparison map display (B). (ns: P > 0. 05, *P < 0. 05, **P < 0. 01, ***P < 0. 001, ****P < 0. 0001). TCGA: The cancer genome atlas; STAD-Stomach cancer; DEGs: differentially expressed genes; GSVA: Gene Set Variation Analysis.

In our analysis of biological responses associated with differentially expressed genes (DEGs) in high and low-risk groups, we compared pathways between these groups and evaluated the activation status of biological pathways using Gene Set Variation Analysis (GSVA). We applied a significance threshold of P. Value < 0. 05, and the most significant 20 pathways were selected for visualization in a heatmap based on log-fold change (logFC) (Fig 7A). The GSVA analysis results for DEGs between high and low-risk groups in the TCGA-STAD dataset revealed significant differences in pathways. Notably, gene sets such as: WALLACE_PROSTATE_CANCER_DN,VALK_AML_WITH_T_8_21_TRANSLOCATION, OHASHI_AURKA_TARGETS, and REACTOME_PHOSPHORYLATION_OF_EMI1 exhibited significant distinctions between high and low-risk groups in the TCGA-STAD dataset. Specifically,WALLACE_PROSTATE_CANCER_DN, VALK_AML_WITH_T_8_21_TRANSLOCATION, and certain other pathways displayed significantly higher enrichment scores in the high-risk group compared to the low-risk group in the TCGA-STAD dataset. Conversely, OHASHI_AURKA_TARGETS, REACTOME_PHOSPHORYLATION_OF_EMI1, and some other pathways exhibited significantly higher enrichment scores in the low-risk group than in the high-risk group within the TCGA-STAD dataset. To visually represent these differential analyses, we used the R package pheatmap to create a heatmap displaying the differential expression of the 20 pathways (Fig 9A). Additionally, we conducted Mann-Whitney U tests to assess the degree of grouping differences among the 20 pathways and presented the results in a group comparison chart (Fig 9B). These analyses provide insights into the specific pathways and biological responses associated with high and low-risk groups in the context of gastric cancer (STAD).

#### Differences in immune signature between high and low risk groups.

**Fig 10:**
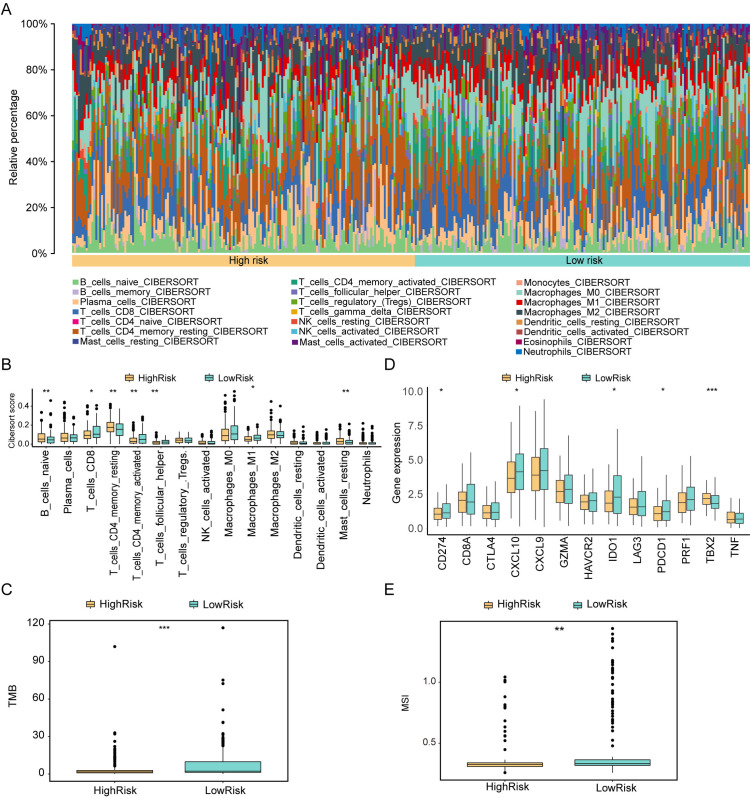
Differences in Immune Signatures Between High and Low-Risk Groups. A. Stacked Diagram of Immune Cell Content in the Tumor Group: Different colors represent distinct immune cell types, and the horizontal axis corresponds to patient IDs. B,D. Box Plots of Immune Cells and Immune Checkpoint Genes in High- and Low-Risk Groups: Yellow represents high-risk group samples, while cyan represents low-risk group samples. C, E. Comparison Graphs of TMB and MSI Values Between High and Low-Risk Groups: TMB (tumor mutational burden) and MSI (microsatellite instability) values are displayed. In all panels, statistical significance is indicated as follows: ns (not significant, p > 0. 05), *p < 0. 05, **p < 0. 01, ***p < 0. 001, ****p < 0. 0001. These Figs illustrate the differences in immune cell infiltration, immune checkpoint gene expression, TMB, and MSI values between high and low-risk groups, providing valuable insights into the immune signatures associated with gastric cancer patients in distinct risk categories.

We evaluated the immune cell infiltration status of 22 different immune cell types using CIBERSORT analysis. To assess the level of immune cell infiltration in gastric cancer patients, we compared the abundance of these immune cell infiltrates using the Wilcoxon test. Additionally, we examined the differences in immune cell infiltration between high and low-risk groups using the CIBERSORT algorithm. Our analysis revealed distinct patterns of immune cell infiltration in high and low-risk groups of gastric cancer patients. Notably, in the high-risk group, certain immune cell types, such as T-cells-CD4-memory-resting, B-cells-naïve, Mast-cells-resting, and others, exhibited significantly higher levels of infiltration compared to the low-risk group (as shown in Fig 10A-10B). These differences in immune cell infiltration were statistically significant (p < 0. 05). Furthermore, our analysis indicated that the low-risk group had higher somatic tumor mutational burden (TMB), suggesting a potentially better response to immunotherapy. Additionally, microsatellite instability (MSI) exhibited significant differences between the high and low-risk groups, with higher MSI observed in the low-risk group (Fig 10C and 10E). In the low-risk group, certain immune checkpoint genes such as CD274, CXCL10, IDO1, and PDCD1 showed higher expression levels (Fig 10D). These findings suggest that the risk group classification may have implications for immune cell infiltration patterns, TMB, MSI, and the potential response to immunotherapy in gastric cancer patients.

#### Impact of Mitophagy Score on Immunotherapy in different datasets.

To assess whether the Risk score of high and low-risk groups holds predictive value for immunotherapy, we conducted a correlation analysis between Risk score and immune infiltrating cells using the GEO dataset. We employed both the ssGSEA algorithm and the CIBERSORT algorithm to evaluate changes and effects in immunological characteristics during the pathogenesis. The results revealed several correlations:Negatively Correlated with Risk Score (p < 0. 05):- Activated-CD4-T-cell (R = −0. 22, −0. 14)- Activated-dendritic-cell (R = −0. 24, −0. 17)- CD56dim-natural-killer-cell (R = −0. 18, −0. 22)- T-cells-CD4-memory-activated (R = −0. 16, −0. 14, −0. 11)- T-cells-CD8 (R = −0. 14, −0. 11)- T-cells-follicular-helper (R = −0. 17, −0. 15, −0. 14)Positively Correlated with Risk Score (p < 0. 01):- Effector-memory-CD4-T-cell (R = 0. 27, 0. 24, 0. 17)- Mast-cell (R = 0. 32, 0. 2)- Mast-cells-resting (R = 0. 18, 0. 14, 0. 13)- T-cells-CD4-memory-resting (R = 0. 2, 0. 17, 0. 13). These correlations, as shown in Fig 10A and 10B, highlight the relationship between Risk score and various immune infiltrating cells, suggesting potential implications for immunotherapy guidance in gastric cancer patients.

#### Construction of clinical prediction model based on mitophagy score.

**Fig 11:**
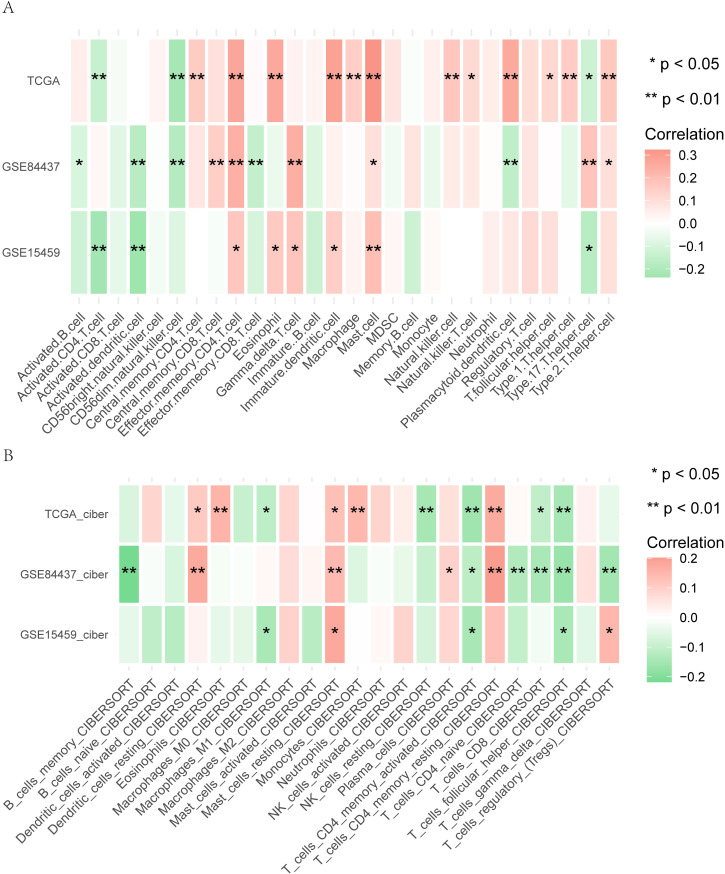
Effect of Mitophagy Score on Immunotherapy for Different Datasets. A, B. Heat maps depicting correlation analysis results between Risk score and immune infiltrating cells, scored using the ssGSEA algorithm and Cibersort algorithm, respectively. The horizontal axis represents various immune infiltrating cells, while the vertical axis represents different datasets. The intensity of the red grid color indicates the strength of the correlation between genes and immune cells, with ‘*’ indicating statistical significance. In both panels, statistical significance is denoted as follows: ns (not significant, p > 0. 05), *p < 0. 05, **p < 0. 01, ***p < 0. 001, ****p < 0. 0001. These heat maps illustrate the correlation between Risk score and immune infiltrating cells, providing insights into the potential impact of Mitophagy Score on immunotherapy across different datasets.

**Fig 12:**
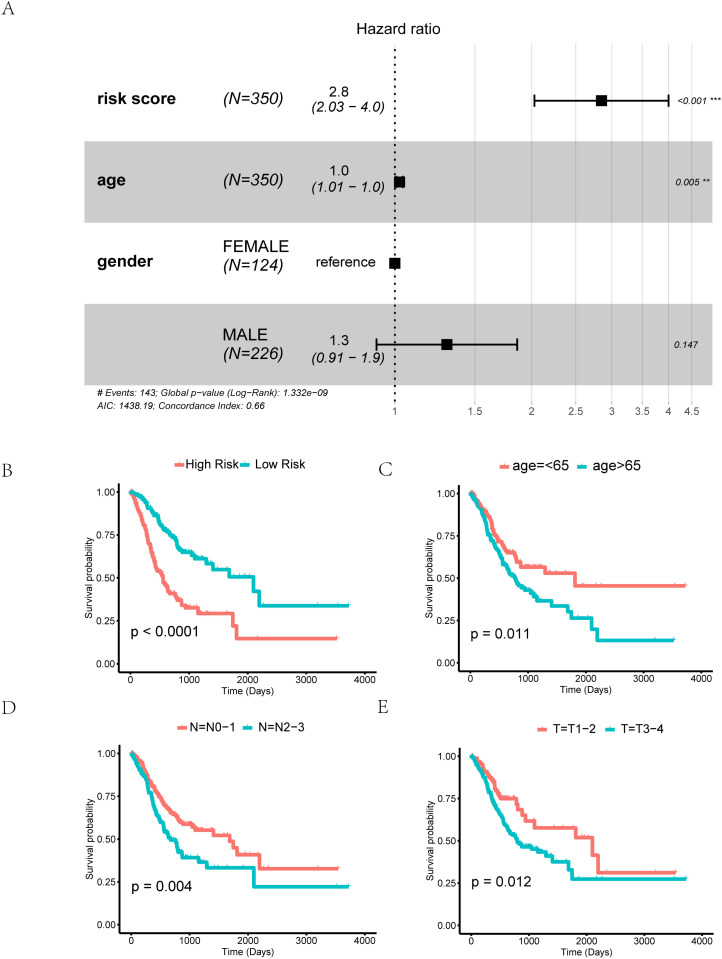
Prognostic Correlation Analysis of Different Risk Factors in GC. A. Forest plots displaying risk score, age, and gender in TCGA-STAD, demonstrating proportional hazards (HR) and P-value (C). B-E. Survival curves depicting high-risk and low-risk groups (B), age (C), stage TN (D-E) in TCGA-STAD.

In order to further explore the clinical potential value of the mitophagy risk score, select the risk score, patient age and patient gender factors to perform multivariate Cox and obtain a forest plot as shown in Fig 12A, the risk score is located on the right side of the invalid line, HR = 2. 8, CI = 2. 03–4. 0, and P < 0. 001. At the same time, we analyzed the clinical characteristics related to the high and low risk groups, such as age, sex and TNM stage prognostic differences. We found that the survival analysis results of high risk and low risk were significantly different (Fig 11B, P < 0. 0001). In addition, the risk score can well distinguish the population older than 60 years old and younger than=<60 years old, stageT1-2 and stageT3-4 population, stageN0-1 and stageN2-3 population (p < 0. 05). (Fig 12B-12E).

**Fig 13:**
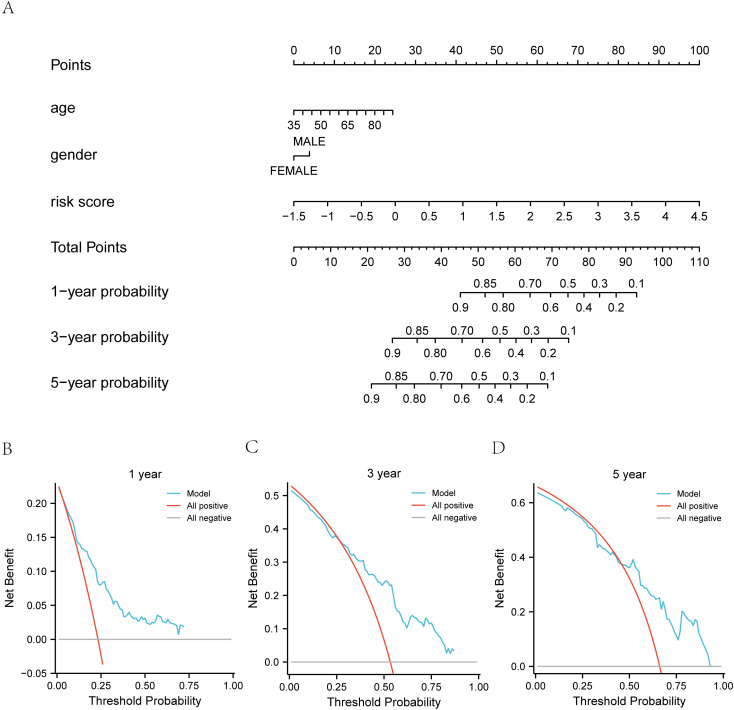
Nomogram and DCA Analysis of Prognostic Assessment. A. Prediction of gastric cancer survival status based on the combination of various factors in the risk score. B-D. Decision curve analysis (DCA) predicting 1-, 3-, and 5-year survival rates for the TCGA-STAD dataset. The x-axis represents the probability threshold or threshold probability (Threshold Probability), while the y-axis represents net benefit. DCA: Decision Curve Analysis. This Fig presents the nomogram for predicting gastric cancer survival and the results of decision curve analysis (DCA) for assessing the clinical utility of the prognostic model at 1, 3, and 5 years in the TCGA-STAD dataset.

Subsequently, we developed a prognostic model nomogram (Fig 12A) based on the Mitophagy Score and clinicopathological characteristics (age, sex, and risk score) of gastric cancer patients. We evaluated the prognostic Cox regression model using decision curve analysis (DCA) to assess its clinical utility at 1 year (Fig 13B), 3 years (Fig 12C), and 5 years (Fig 13D). The results are presented in Fig 16B-16D. In the DCA graph, the x-axis represents the probability threshold or threshold probability, and the y-axis represents net benefit. The model’s effectiveness is judged based on the x-value range where the model’s curve remains consistently higher than both the All-positive and All-negative lines. A larger x-value range indicates a better model performance. The results demonstrate that the Cox regression prognostic model we constructed is effective, with the clinical prediction effect ranking as 3 years > 1 year > 5 years.

#### Knockdown of DUSP1 suppress cell proliferation and migration in GC.

**Fig 14:**
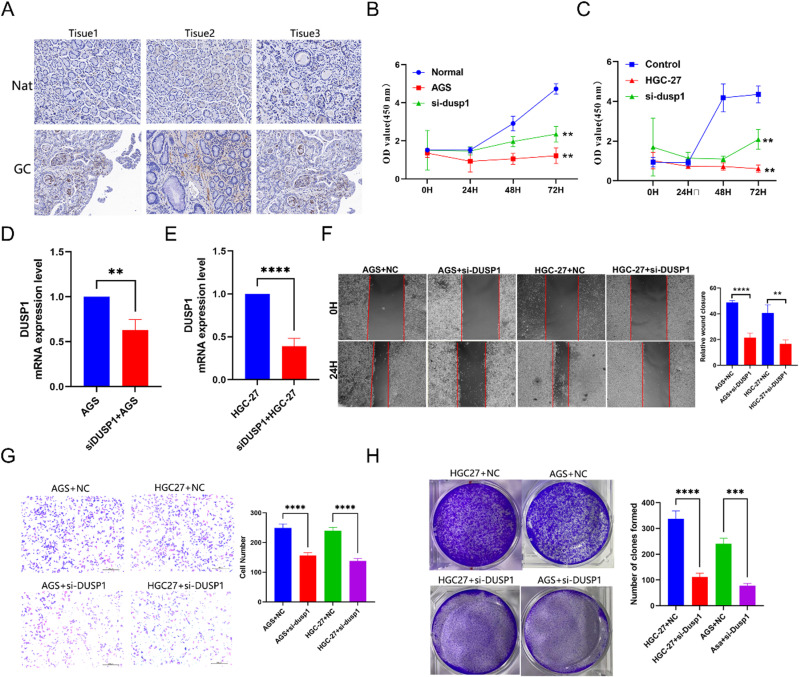
DUSP1 expression and effect on proliferation and migration in GC. A. Immunohistochemistry detection of the DUSP1 in the GC and normal adjacent tissue. B-C. Knockdown of DUSP1 in GC cells and validated the efficiency of si-DUSP1 and si-Control (NC) transfection in GC cells proliferation by qPCR. D-E. Knockdown of DUSP1 in GC cells and validated the efficiency of si-DUSP1 and si-Control (NC) transfection in GC cells proliferation by CCK-8. F. Wound healing assays of cell migration in GC cells. The images of wound closure are presented at the indicated number of hours after scratching (0, 24 h). G. Transwell assays were performed to examine the potential migration of si-DUSP1 cells or negative control cells. H. Colony formation assay were performed to examine proliferation of si-DUSP1 cells and si-NC of GC. Additional information is in S1 File.

Immunostaining of normal tissue adjacent to cancer and tumor tissue found that DUSP1 highly expressed in GC (Fig 14A). Our experiments confirm si-DUSP1 can downregulate this miRNA expression of this gene (Fig 14D-14E). Then, We conducted the CCK-8 and colony formation assay, compared to the NC group, the group of si-DUSP1 can significant decrease in cell viability and the number of clones forming. (FigB. C. H). From this we conclude that knock-down DUSP1 inhibits cell proliferation of GC cells. From this we conclude that knock-down DUSP1 inhibits cell proliferation of GC cells. Lastly, we conducted the scratch migration and transwell assay to determine migration capacity after knock-down DUSP1. Result in there picture (Fig. F-G) showing knock-down DUSP1 diminishes migration capacity of GC cells.

## Discussion

In recent years, mitochondrial autophagy (mitophagy) has garnered significant attention from researchers due to its unique biological functions in tumors. Studies have demonstrated that mitophagy plays complex and multifaceted roles in tumor initiation and progression. It can potentially suppress tumor cell proliferation, while also exacerbating tumor progression by promoting drug resistance and metastatic capabilities of tumor cells. Mitophagy is critically involved in tumorigenesis, metastasis, recurrence, and resistance to chemotherapy and radiotherapy [29]. However, the pathophysiological roles and mechanisms of mitochondrial autophagy in gastric cancer still require further exploration. Therefore, this article utilizes bioinformatics analysis to investigate the roles of mitophagy-related genes in gastric cancer. It explores the molecular characteristics of these genes and conducts experimental validation of key molecules to determine their roles in the progression of gastric cancer.

Through multi-omics screening, we identified 22 autophagy-related genes and assessed their chromosomal positions and somatic mutations. Further analysis using the Protein-Protein Interaction (PPI) network pinpointed core molecules among these genes as HIF1A, BCL2L1, and PARK2. Additionally, by constructing a ceRNA regulatory network, we delved deeper into the role of molecular networks in gastric cancer. We also developed a risk score related to mitochondrial autophagy genes, discovering that patients with a high-risk score exhibited poorer survival, thus providing a new pathway for predicting the prognosis of gastric cancer patients. Research has demonstrated that pathways such as Wnt, MAPK, and Phosphatidylinositol-3-kinase (PI3K)/AKT/mammalian target of rapamycin (mTOR) can promote tumor development and progression. We observed that the group with a high mitochondrial autophagy-related gene risk score was significantly enriched in these pathways [30–32]. In summary, we believe that a high mitochondrial autophagy-related gene risk score can reflect characteristics of gastric cancer progression.

In this study, a mitochondrial autophagy-related risk score was developed, encompassing eight genes. Through single-cell analysis, we further demonstrated the expression profiles of these eight genes across different cell types. Notably, DUSP1 expression was significantly elevated in various cell types within gastric cancer tissues, suggesting that DUSP1 may play a crucial role in the progression of gastric cancer. As an important signaling regulator, DUSP1 is involved in key processes such as tumor cell proliferation, apoptosis, and drug resistance [5,33]. The expression levels of DUSP1 are closely associated with the clinical prognosis of various cancers. In this study, we analyzed the expression of DUSP1 in gastric cancer and found that it was significantly higher in tumor tissues compared to normal tissues. Additionally, knockdown of DUSP1 significantly inhibited the proliferation and migration of gastric cancer cells. These results further suggest that DUSP1 may promote the development and progression of gastric cancer.

In this study, we developed a mitochondrial autophagy-related risk score and systematically described its association with tumor microenvironment (TME) immune cell infiltration and tumor mutational burden (TMB). The results demonstrated that in gastric cancer patients, there exist distinct high and low-risk groups based on mitochondrial autophagy-related genes, which play a significant role in the tumorigenesis, progression, and treatment of cancer. The mitochondrial autophagy-related risk score may provide new insights for subsequent patient survival assessment, guide personalized treatment, and enhance the efficacy of immunotherapy. This conclusion has explored the cell signaling cross-over between memory B cells and tumor cells in nasopharyngeal carcinoma, and it is thought to have an impact on the prognosis of tumors [34]. However, inevitably, our study also has limitations. Firstly, the data used in our study are sourced from public databases, lacking proprietary data. Although cellular experiments were validated, further expansion of experimental sample size is needed to verify the accuracy of the experiments. Additionally, we only investigated the role of the core gene DUSP1 in gastric cancer, and the functional experiments of other genes in the model need further validation to explore the role and mechanisms of autophagy-related genes in gastric cancer. The mechanisms of gene interactions targeting mitochondrial autophagy related risk in gastric cancer are also worth exploring. Secondly, the lack of clinical data verification necessitates obtaining large samples from cooperating hospitals in the future to validate the accuracy of mitochondrial autophagy-related findings. Lastly, further in-depth clinical studies are required to explore the actual role of mitochondrial autophagy in immunotherapy. We look forward to future studies on the clinical relevance of dusp1, such as whether there is a synergistic effect between DUSP1 and our chemotherapy and immunity. How to combine mitophagy-related gene risk scores with tumor mutation burden, PDL1 expression and other factors to guide individual treatment is also worth exploring.

## Supporting information

Table S1 Mitophagy-related genes by compiling a list of 489 candidate genes sourced. from both the GeneCards database and the GSEA database.
(DOCX)

Table S2 Analysis yielded 14 PPI nodes (proteins) and 84 connecting edges. (DOCX)

S1 File Raw data of Cell experiments. (ZIP)
