# Supplementary material for: Utilizing Multi-omics analysis to elucidate the role of mitochondrial gene defects in Gastric cancer progression
Source: PLoS One. 2025 Jun 9;20(6):e0325520. doi: 10.1371/journal.pone.0325520 (PMC12148105; doi:10.1371/journal.pone.0325520)
Supplement: Table S1 — from both the GeneCards database and the GSEA database. (DOCX) [file pone.0325520.s001.docx]

**Table 1 Baseline data table**

| Characteristic | levels | Overall |
| --- | --- | --- |
| n |  | 375 |
| T stage, n (%) | T1 | 19 (5.2%) |
|  | T2 | 80 (21.8%) |
|  | T3 | 168 (45.8%) |
|  | T4 | 100 (27.2%) |
| N stage, n (%) | N0 | 111 (31.1%) |
|  | N1 | 97 (27.2%) |
|  | N2 | 75 (21%) |
|  | N3 | 74 (20.7%) |
| M stage, n (%) | M0 | 330 (93%) |
|  | M1 | 25 (7%) |
| Age, median (IQR) |  | 67 (58, 73) |
