# Supplementary material for: Utilizing Multi-omics analysis to elucidate the role of mitochondrial gene defects in Gastric cancer progression
Source: PLoS One. 2025 Jun 9;20(6):e0325520. doi: 10.1371/journal.pone.0325520 (PMC12148105; doi:10.1371/journal.pone.0325520)
Supplement: Table S2 — (DOCX) [file pone.0325520.s002.docx]

**Table 2 GO analysis**

| **Description** | **pvalue** | **p.adjust** |
| --- | --- | --- |
| cell-cell adhesion via plasma-membrane adhesion molecules | 9.9188E-32 | 5.9265E-28 |
| homophilic cell adhesion via plasma membrane adhesion molecules | 4.2382E-31 | 1.2662E-27 |
| muscle contraction | 1.4782E-29 | 2.944E-26 |
| modulation of chemical synaptic transmission | 2.8381E-28 | 4.2395E-25 |
| regulation of trans-synaptic signaling | 3.7765E-28 | 4.5129E-25 |
| muscle system process | 7.2485E-27 | 7.2183E-24 |
| synapse organization | 1.8399E-26 | 1.5705E-23 |
| regulation of membrane potential | 2.0865E-24 | 1.5583E-21 |
| synapse assembly | 1.1087E-23 | 7.3608E-21 |
| regulation of blood circulation | 6.0353E-23 | 3.6061E-20 |
| multicellular organismal signaling | 9.7198E-23 | 5.2796E-20 |
| extracellular structure organization | 2.3274E-21 | 1.1588E-18 |
| heart contraction | 8.7309E-21 | 4.0129E-18 |
| extracellular matrix organization | 1.7865E-20 | 7.6245E-18 |
| axonogenesis | 2.1483E-20 | 8.5574E-18 |
| heart process | 5.0291E-20 | 1.8781E-17 |
| regulation of ion transmembrane transport | 7.4325E-20 | 2.6123E-17 |
| regulation of heart contraction | 1.0641E-19 | 3.5323E-17 |
| neuron projection guidance | 1.8923E-15 | 5.9508E-13 |
